# A Two-Component Regulatory System in Transcriptional Control of Photosystem Stoichiometry: Redox-Dependent and Sodium Ion-Dependent Phosphoryl Transfer from Cyanobacterial Histidine Kinase Hik2 to Response Regulators Rre1 and RppA

**DOI:** 10.3389/fpls.2016.00137

**Published:** 2016-02-12

**Authors:** Iskander M. Ibrahim, Sujith Puthiyaveetil, John F. Allen

**Affiliations:** ^1^Faculty of Engineering and Science, University of Greenwich, Chatham MaritimeKent, UK; ^2^Institute of Biological Chemistry, Washington State UniversityPullman, WA, USA; ^3^Research Department of Genetics, Evolution and Environment, University College LondonLondon, UK

**Keywords:** redox sensor, redox regulator, photosystem stoichiometry, transcriptional control, Histidine Kinase 2, Chloroplast Sensor Kinase (CSK), *Synechocystis sp*. PCC 6803, salt stress

## Abstract

Two-component systems (TCSs) are ubiquitous signaling units found in prokaryotes. A TCS consists of a sensor histidine kinase and a response regulator protein as signal transducers. These regulatory systems mediate acclimation to various environmental changes by coupling environmental cues to gene expression. Hik2 is a sensor histidine kinase and its gene is found in all cyanobacteria. Hik2 is the homolog of Chloroplast Sensor Kinase (CSK), a protein involved in redox regulation of chloroplast gene expression during changes in light quality in plants and algae. Here we describe biochemical characterization of the signaling mechanism of Hik2 and its phosphotransferase activity. Results presented here indicate that Hik2 undergoes autophosphorylation on a conserved histidine residue, and becomes rapidly dephosphorylated by the action of response regulators Rre1 and RppA. We also show that the autophosphorylation of Hik2 is specifically inhibited by sodium ions.

## Introduction

Bacteria are found in almost every habitable environment, and successfully adapt to environmental change in a wide range of different ecological niches. One reason for the ecological success of bacteria is their remarkable ability to sense and respond to changing environmental conditions. For this environmental acclimation, bacteria mostly utilize sensor-response circuits known as two-component systems (TCSs). TCSs mediate acclimatory responses by changing bacterial cellular physiology, which is accomplished in most cases by regulation of gene expression at transcriptional and post-transcriptional levels. Each TCS consists of two proteins, a sensor histidine kinase (component 1) and a response regulator (component 2) (Stock et al., 2000). Upon environmental stimulus, the sensor histidine kinase undergoes autophosphorylation on the conserved histidine residue, receiving the γ-phosphate from ATP. The phosphoryl group from the histidine is subsequently transferred to a conserved aspartate residue in the response regulator to cause a structural change that elicits a change in target gene expression (Stock et al., 2000).

Although TCSs are ubiquitous in bacteria, in eukaryotes they are found only in plants, fungi, and protists. Cyanobacterial genomes typically encode a large number of two-component systems, ranging from as many as 146 histidine kinases and 168 response regulators in the filamentous cyanobacterium *Nostoc punctiforme* to as few as five histidine kinases and six response regulators in the small genome of the marine unicellular cyanobacterium *Prochlorococcus* MED4 (Mary and Vaulot, 2003). Three histidine kinases are fully conserved in all cyanobacterial genomes. One of these is Histidine kinase 2 (Hik2) (Ashby and Houmard, 2006). Interestingly, a homolog of Hik2 is also found in chloroplasts of nearly all algae and plants as Chloroplast Sensor Kinase (CSK). This wide distribution of Hik2 and CSK suggests important functional roles for these sensors in cyanobacteria and chloroplasts, respectively (Puthiyaveetil et al., 2008). In chloroplasts of the model plant *Arabidopsis thaliana*, CSK regulates transcription of chloroplast genes in response to changes in reduction-oxidation (redox) potential of the photosynthetic electron transport chain (Puthiyaveetil et al., 2008). *csk* knockout plants are unable to link changes in light quality to the expression of photosynthetic reaction center genes in chloroplast DNA. Therefore, the CSK-signaling pathway has been suggested to underlie the acclimatory process of adjustment of the stoichiometry of chloroplast photosystem I and photosystem II (Puthiyaveetil et al., 2008).

A yeast two-hybrid analysis of cyanobacterial two-component systems demonstrates interaction of Hik2 with Response regulator 1 (Rre1) (Sato et al., 2007). A homolog of Rre1 also occurs in chloroplasts of non-green algae as Ycf29 (hypothetical chloroplast open reading frame 29; Puthiyaveetil et al., 2008; Puthiyaveetil and Allen, 2009). In non-green algae, CSK is likely to regulate chloroplast genes through Ycf29 by means of the His-to-Asp phosphotransfer mechanism. However, in green algae and higher plants, Ycf29 has been lost, and CSK regulates transcription of chloroplast genes through phosphorylation of chloroplast sigma factor 1 (SIG1) in a catalytic mechanism similar to that of serine/threonine kinases (Puthiyaveetil et al., 2010, 2012, 2013). This rewiring of the CSK signaling pathway may have accompanied replacement of the original, conserved histidine residue in plant and green algal CSKs.

**Figure 1:**
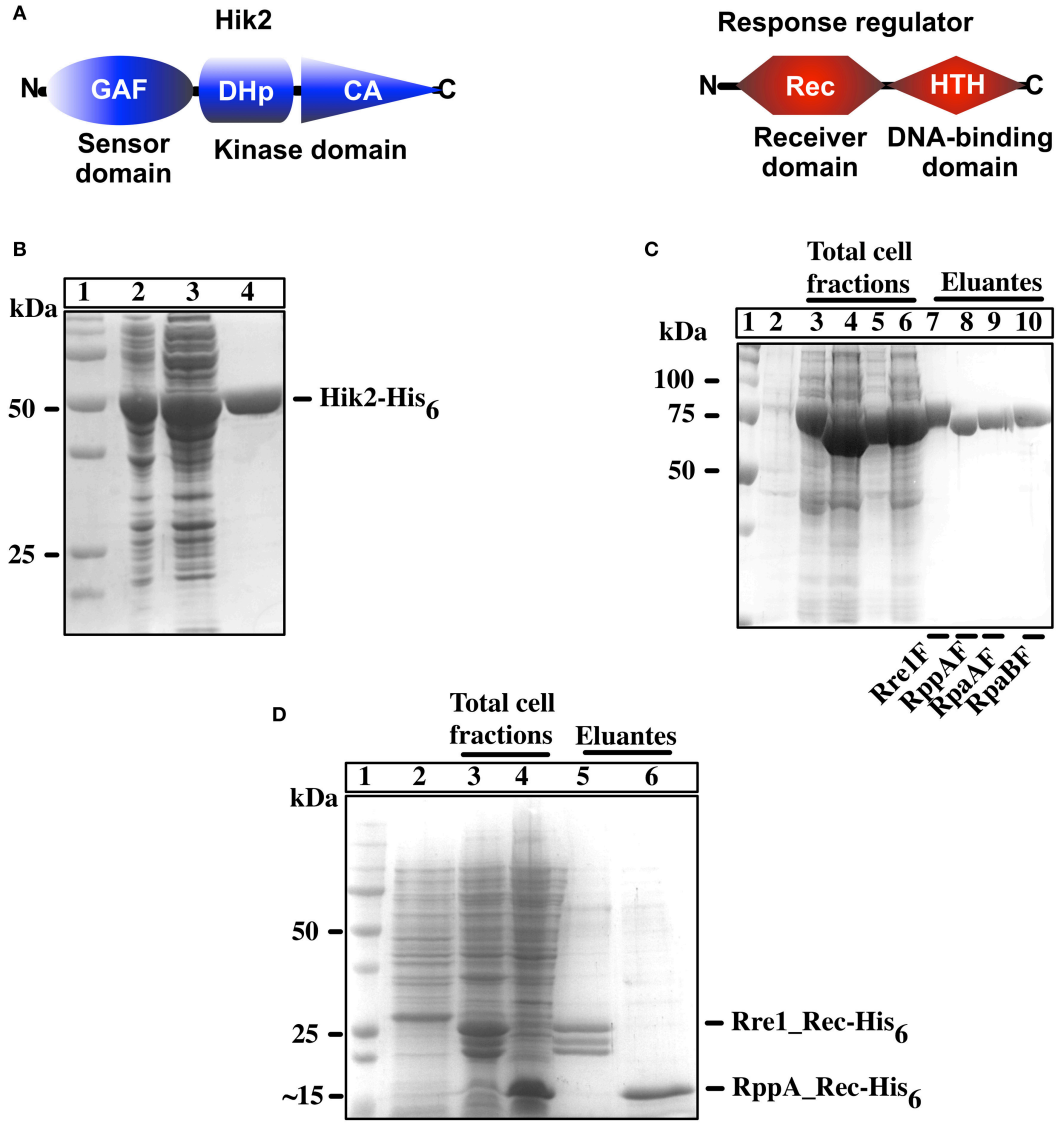
**Overexpression and purification of recombinant Hik2 and response regulators**. **(A)** Schematic representation of domain architecture of full-length Hik2 and its putative response regulators as predicted by the SMART database (Chenna et al., 2003). The GAF (named after its presence in cGMP-specific phosphodiesterases, in certain Adenylyl cyclases, and in transcription factor FhlA) domain and the conserved DHp (dimerization and phosphoacceptor) and CA (Catalytic and ATP-binding) domains of Hik2 are depicted by blue oval, cylinder, and triangle, respectively. The conserved receiver (Rec) and the helix-turn-helix (HTH) DNA-binding domains of response regulator are depicted in red hexagon and parallelogram, respectively. **(B)** Protein overexpression and purification for Hik2F. Different cell fractions separated on a 12% SDS-PAGE and stained with Coomassie brilliant blue are shown. Lane 1 shows protein molecular weight standards in kDa; in lane 2 is total cell fraction after IPTG induction; lane 3 is soluble cell fraction; lane 4 is purified Hik2 protein. **(C)** Full-length response regulators: protein overexpression and purification. In lane 1 are protein molecular weight standards identified numerically in kDa; in lane 2 is the total cell fraction before IPTG induction; lanes 3–6 are total cell fractions containing N-terminus MBP-tagged Rre1F (lane 3), RppAF (lane 4), RpaAF (lane 5), RpaB (lane 6); lanes 7–10 are purified proteins; Rre1F (lane 7), RppAF (lane 8), RpaAF (lane 9), and RpaBF (lane 10). **(D)** Receiver domain of Rre1 and RppA: lane 1 shows molecular weight marker; lane 2 is total cell fraction before IPTG induction; lanes 2 and 3 are total cell fraction after IPTG induction containing receiver domain of Rre1 (Rre1_Rec, lane 3), and RppA (RppA_Rec, lane 4); lanes 5 and 6 are purified proteins; Rre1_Rec (lane 5) and RppA_Rec (lane 6). The positions of molecular weight markers are indicated on the left and their values given in kDa.

Hik2, in contrast to CSK, contains all motifs characteristic of bacterial histidine kinases, including the conserved histidine residue. Hik2 also has a clearly identifiable GAF sensor domain at its N-terminus (Figure 1A). Hik2, like CSK, does not contain transmembrane helices and is probably a soluble sensor kinase. However, little is known about the precise functional role of Hik2 and its signaling mechanism. Interestingly, in addition to Rre1, a second response regulator RppA (Regulator of photosynthesis and photopigment-related gene expression A) also interacts with Hik2 in a yeast two-hybrid assay (Sato et al., 2007). RppA is a redox response regulator that regulates photosynthesis genes (Li and Sherman, 2000). In cyanobacteria, photosynthesis genes are also regulated by a paralogous group of response regulators known as RpaA and RpaB (Regulator of phycobilisome association; Ashby and Mullineaux, 1999; Kato et al., 2011; Majeed et al., 2012). Homologs of RpaB are also found in the chloroplasts of some non-green algae (Ashby et al., 2002; Puthiyaveetil and Allen, 2009). It has been unclear whether Hik2 interacts with RpaA or RpaB. Hik2 has also been implicated in osmosensing, raising the possibility that it is a multi-sensor kinase (Paithoonrangsarid et al., 2004). However, direct evidence for signals affecting Hik2 activity has been lacking thus far.

In laboratory conditions, most genes coding for two-component regulatory proteins can be inactivated in cyanobacteria without adverse effect on cell growth. However, the *Hik2* gene seems to be indispensable (Paithoonrangsarid et al., 2004). In order to understand the signaling and functional properties of Hik2, we employed an *in vitro* approach. The sensory mechanism of Hik2 and its interaction with its putative response regulators are explored in this study. Here, we demonstrate autophosphorylation of Hik2 on a conserved histidine residue, and show that this autophosphorylation depends on the presence of glycine residues in the protein's ATP-binding domain. We also find that autophosphorylation is specifically inhibited in the presence of NaCl. Furthermore, we show rapid and specific phosphotransfer activity from Hik2 to both Rre1 and RppA, thus identifying them as genuine response regulators and functional interaction partners of Hik2 in cyanobacteria. The interaction of Hik2 with Rre1 and RppA is further confirmed by a pull-down assay. Our results suggest that a Hik2-based signaling pathway integrates acclimatory responses to light and salt stresses in cyanobacteria.

## Materials and methods

### Construction of recombinant plasmids

Coding sequences corresponding to the full-length *Synechocystis sp*. PCC 6803 Hik2 (slr1147), Rre1 (slr1783), RppA (sll0797), RpaA (sll0797), and to the receiver domains of response regulators Rre1 and RppA were amplified from *Synechocystis* sp. PCC 6803 genomic DNA using the primer pairs listed in Table 1. PCR products of full-length Hik2 (Hik2_F) was digested with *NdeI* and *XhoI* or *BamH* and *XhoI* endonucleases (New England BioLabs) and cloned into a pET-21b (Invitrogen) or pETG-30A (EMBL) expression vectors. PCR products of full-length Rre1, RppA, RpaA, and RpaB were digested with *KpnI* and *XhoI* endonucleases (New England BioLabs) and cloned into pETG-41A (EMBL) expression vector. PCR products of receiver domains of Rre1 and RppA were digested with *BamHI* and *XhoI* and cloned into pET-30a(+) expression vector (Invitrogen). The identities of the recombinant clones were confirmed by sequencing (results not shown).

**Table 1:** **Primer pairs used for cloning *Hik2, Rre1, RppA, RpaA*, and *RpaB***.

– Hik2F_His_6_ (cloned in pET-21b) Forward: GCGCGCcatatgGCCGGTTCCATCTCA Reverse: GCGCGCctcgagCACTTGTTCTCCAGAGCG
– Hik2F_GST (cloned in pETG-30A) Forward: TTGGCGggtaccATGGCCGGTTCCATCTCA Reverse: GCGCGCctcgagCACTTGTTCTCCAGAGCG
– Rre1F_MBP (cloned into pETG-41A) Forward: GCGCGCggtaccGTGGGCTTGAGTTTGCTG Reverse: GCGGCGctcgagCTAGACGATCGCCTCCAATTC
– RppAF_MBP (cloned into pETG-41A) Forward: GCGCGCggtaccCGAATTTTGCTGGTGGAA Reverse: GCGGCGctcgagCTACAGTCTTGCTAATAGCTC
– RpaAF_MBP (cloned into pETG-41A) Forward GCGCggtaccATGCCTCGAATACTGATC Reverse: GCGCGCctcgagCTACGTTGGACTACCGCC
– RpaBF_MBP (cloned into pETG-41A) Forward: GCGCGCggtaccGTGGTCGATGACGAGGCC Reverse: GCGGCGctcgagCTAGATTCTAGCTTCCAATTC
– Rre1_Receiver_His_6_ (cloned into pET-30a+) Forward: GCGGCGggatccATGGTGGGCTTGAGTTTG Reverse: GCGGCGctcgagCTAGACGATCGCCTCCAATTC
– RppA_Receiver_His_6_ (cloned into pET-30a+) Forward: GCGGCGggatccATGCGAATTTTGCTGGTG Reverse: GCGGCGctcgagCTACAGTCTTGCTAATAGCTC
For H-box
–His^185^Q Forward: CTGACCTCTTGCAGCAACTCCGCAATC Reverse: GATTGCGGAGTTGCTGCAAGAGGTCAG
For G1-box
– Gly^359^A Forward: CGCCGACACGGCTTATGGCATTC Reverse: GAATGCCATAAGCCGTGTCGGCG
– Gly^361^A Forward: GATCGCCGACACGGGTTATGCGATTCCCCCGGAGGATCAAC Reverse: GTTGATCCTCCGGGGGAATTGCATAACCCGTGTCGGCGATC
For G2-box
– Gly^386^A Forward: CGAGGCTCCATTAATGCGACTGGTTTGGGTTTG Reverse: CAAACCCAAACCAGTCGCATTAATGGAGCCTCG
– Gly^388^A Forward: CATTAATGGCACTGCGTTGGGTTTGGCGATC Reverse: GATCGCCAAACCCAACGCAGTGCCATTAATG
– Gly^390^A Forward: CACTGGTTTGGCATTGGCGATCGTG Reverse: CACGATCGCCAATGCCAAACCAGTG

*Sequences in lower case are restriction site overhangs. Sequences underlined are codons for glutamine or alanine*.

### Site-directed mutagenesis of the conserved motifs within H, G1, G2, boxes of Hik2

Mutagenesis of the conserved histidine residue of the H-box (His^185^) to glutamine, and of the conserved glycine residues of the G1 box (Gly^359^ and Gly^361^), and G2-box (Gly^386^, Gly^388^, and Gly^390^) to alanine was made using Stratagene QuickChange site-directed mutagenesis kit. The primer pairs used are listed in Table 1. Mutagenesis was confirmed by sequencing (results not shown).

### Expression and purification of recombinant Hik2 and response regulators

Recombinant plasmids were transformed into *E. coli* BL21(DE3) chemically competent cells (Stratagene). Transformed bacterial colonies, grown on agar plates, were used to inoculate starter cultures (10mL each) in Luria Broth (LB) growth media (Sambrook et al., 1989) with 100μg mL^−1^ ampicillin for the Hik2 and full-length response regulator clones, or with 35μg mL^−1^ kanamycin for clones containing receiver domain response regulators, as the selectable marker. Each culture was grown overnight, then diluted 1:100 in 1 L LB media and grown at 37°C to an optical density at 600nm of ~0.55, before inducing protein expression with 0.5mM IPTG (Melford). Bacterial cultures were grown for a further 16 h at 16°C. Cells were harvested by centrifugation at 6000 rpm for 10 min. The pellet was re-suspended in a buffer containing 300mM NaCl, 20mM Tris-HCl, pH 7.4, 25mM imidazole, and 1mM PMSF, and the cells lysed with an EmulsiFlex-C3 homogenizer (Avestin). Lysate was separated by centrifugation at 18,000 rpm for 20 min. The supernatant was applied to a Ni^2+^ affinity chromatography column (GE Healthcare) and the C-terminally poly-histidine tagged Hik2 protein, the N-terminally poly-histidine tagged receiver domain of response regulators, and also the full-length response regulators obtained from a pETG-41A vector containing an N-terminal poly-histidine tag followed by a MPB tag were all purified using a Ni^2+^ affinity chromatography column according to the column manufacturer's instructions. For the salt treatment assay, full-length Hik2 protein was desalted into Tris-HCl (10mM final, pH 7.4) using PD-10 desalting column (Amersham Biosciences) and used in the autophosphorylation assay immediately.

### Pull-down assay

The bait and prey proteins were overexpressed as described above. Bacterial pellets containing the overexpressed proteins were re-suspended in 5mL of phosphate-buffered saline (PBS) (140mM NaCl, 2.7mM KCl, 1.8mM KHPO_4_, and 8.1mM NaHPO_4_ at pH 7.3) and lysed by several freeze-thaw cycles, followed by sonication three times for 15 s at maximum power. The lysate was clarified by centrifugation at 18,000 rpm for 20 min. The supernatant containing bait proteins (Hik2-GST or GST) was incubated with Protino Glutathione Agarose 4B particles (Promega) and washed six times with 10-bead volume of ice-cold PBS. Prey (Rre1_Rec-His_6_ and RppA_Rec-His_6_) and bait (Hik2-GST or GST) proteins were then mixed together and incubated for 2 h at 19°C on a rotating platform. The supernatant was removed and the pelleted-beads were washed 3–4 times and eluted according to the manufacturer's instructions.

### *In vitro* autophosphorylation assay

Autophosphorylation was performed with 2μM of purified recombinant Hik2 protein in a kinase reaction buffer (50mM Tris-HCl (pH 7.5), 50mM KCl, 10% glycerol, and 10mM MgCl_2_) in a final reaction volume of 25μL. The autophosphorylation reaction was initiated by the addition of 5μL of five-fold concentrated ATP solution containing 2.5mM disodium ATP (Sigma) with 2.5μCi [γ-^32^P]-ATP (6000 Ci mmol^−1^) (PerkinElmer) or with 5μCi [α-^32^P]-ATP (3000 Ci mmol^−1^) as a control. Reactions were incubated for 15 s at 22°C. The autophosphorylation reaction was terminated by addition of 6μL of five-fold concentrated Laemmli sample buffer (Laemmli, 1970). Reaction products were resolved on a 12% SDS-PAGE (sodium dodecyl sulfate polyacrylamide gel electrophoresis) gel. The gel was rinsed with SDS running buffer and transferred into a polyethylene bag. The sealed bag was exposed to a phosphor plate overnight. The incorporated γ-^32^P was visualized using autoradiography and the band intensity from the autoradiograph was quantified using ImageJ version 1.44 (Schneider et al., 2012).

### Autophosphorylation assay in the presence of salt

2μM recombinant Hik2 protein was pre-equilibrated with 5μL of five-fold concentrated, low potassium reaction buffer [250mM Tris-HCl (pH 7.5), 25mM KCl, 50% glycerol, and 50mM MgCl_2_] and with water, as a control, or with the following salts: NaCl (0.3M final concentration), Na_2_SO_4_ (0.25M final concentration), NaNO_3_ (0.3 M final concentration), or KCl (0.375 M final concentration) in a total reaction volume of 20μL. Reaction mixtures were then incubated at room temperature (22°C) for 30 min. Autophosphorylation was assayed as above. Hik2 was titrated with varying concentrations of NaCl and autophosphorylation was performed for 15 s. The incorporated γ-^32^P was visualized and the band intensity quantified as described earlier. The concentration-dependent inhibition curve for Hik2 was plotted from data points representing at least three independent experiments, using Prism 6 (Motulsky and Christopoulos, 2003).

### Acid-base stability assay

Four replicates of autophosphorylation reactions of Hik2 were performed as above. Proteins were then resolved on a 12% SDS-PAGE gel and blotted onto a PVDF membrane. Each lane containing the autophosphorylated Hik2 protein was excised and incubated in 50–100 mL of 50mM Tris-HCl (pH 7.4; neutral conditions), 1 M HCl (acidic conditions), or 3 M NaOH (basic conditions) for 2.5 h at 55°C with agitation. The extent of γ-^32^P hydrolysis was analyzed using autoradiography.

### Phosphotransfer analysis

The autophosphorylation reaction was carried out by mixing 30μM Hik2 in a total reaction volume of 375μL containing kinase reaction buffer [50mM Tris-HCl (pH 7.5), 50mM KCl, 10% glycerol, 25mM MgCl_2_, and 2mM DTT] and ATP [2.5mM disodium ATP and 37.5μCi [γ-^32^P]ATP (6000 Ci mmol^−1^)]. The reaction mixture was incubated at 30°C for 10min. In the meantime, 25μM of each of the response regulators Rre1, RppA, RpaA, or RpaB were diluted with the kinase reaction buffer to give a total volume of 62.5μL. A control lacking response regulator was prepared in the same way, except that response regulator protein solution was replaced with an equal volume of water. For each phosphotransfer reaction, 62.5μL of autophosphorylated radiolabeled Hik2 protein was mixed with 62.5μL of the response regulator or with the water control. Kinase and response regulator were present at a concentration of 1 and 5μM, respectively. Reactions were mixed and incubated at 30°C. Twenty-five microliter samples were removed at 0, 20, 40, 60, and 90min, and the reactions stopped by the addition of Laemmli sample buffer. Proteins were resolved on 15% SDS-PAGE and the presence of γ-^32^P was analyzed using autoradiography. The incorporated γ^32−^P was visualized and quantified as before.

## Results

### Overexpression and purification of full-length Hik2 and response regulators recombinant proteins

In order to examine the autophosphorylation activity of full-length Hik2 protein and its interaction with its putative response regulator(s), we cloned the coding sequences of full-length *Synechocystis* sp. PCC 6803 *Hik2, Rre1, RppA*, and *RpaA and RpaB* genes. Figure 1 shows the purified C-terminally His_6_ tagged full-length Hik2 (Figure 1B); N-terminally His_6_-MBP tagged full-length response regulators (Figure 1C) and N-terminally His_6_ tagged receiver domains (Figure 1D) separated on a reducing SDS-PAGE gel. The apparent molecular weights are: Hik2, 50 kDa (Figure 1B, lane 4); Rre1F, 75 kDa (Figure 1C, lane 7); RppAF, 70 kDa (Figure 1C, lane 8); RpaAF, 72 kDa (Figure 1C, lane 9); and RpaBF, 72 kDa (Figure 1C, lane 10); Rre1_Rec, 25 kDa (Figure 1D, lane 5); and RppA_Rec 15 kDa, (Figure 1D, lane 6). Theoretical molecular weights are: Hik2, 49 kDa; Rre1F, 75 kDa; RppAF, 70 kDa; RpaAF, 71 kDa; and RpaBF, 70 kDa; Rre1_Rec, 20 kDa; and RppA_Rec 14 kDa.

### The full-length Hik2 autophosphorylates *In vitro;* Na^+^ ions inhibit its autophosphorylation activity

**Figure 2:**
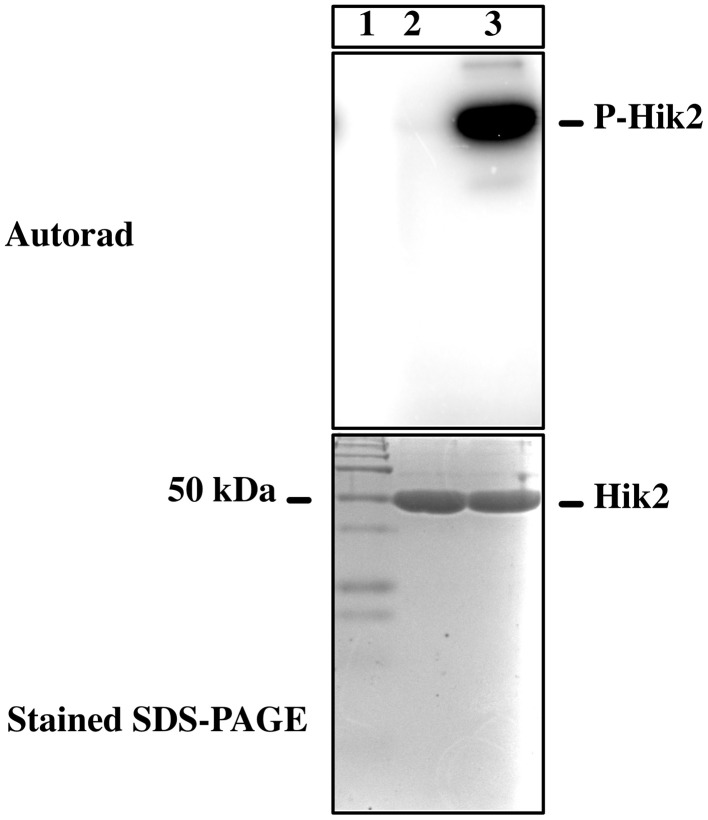
**Autophosphorylation activity of Hik2**. An autoradiograph of the reaction product is presented. Lane 1 is protein molecular weight marker in kDa; in lane 2 Hik2 was incubated with [α-^32^P]-ATP; lane 3, with [γ-^32^P]-ATP.

**Figure 3:**
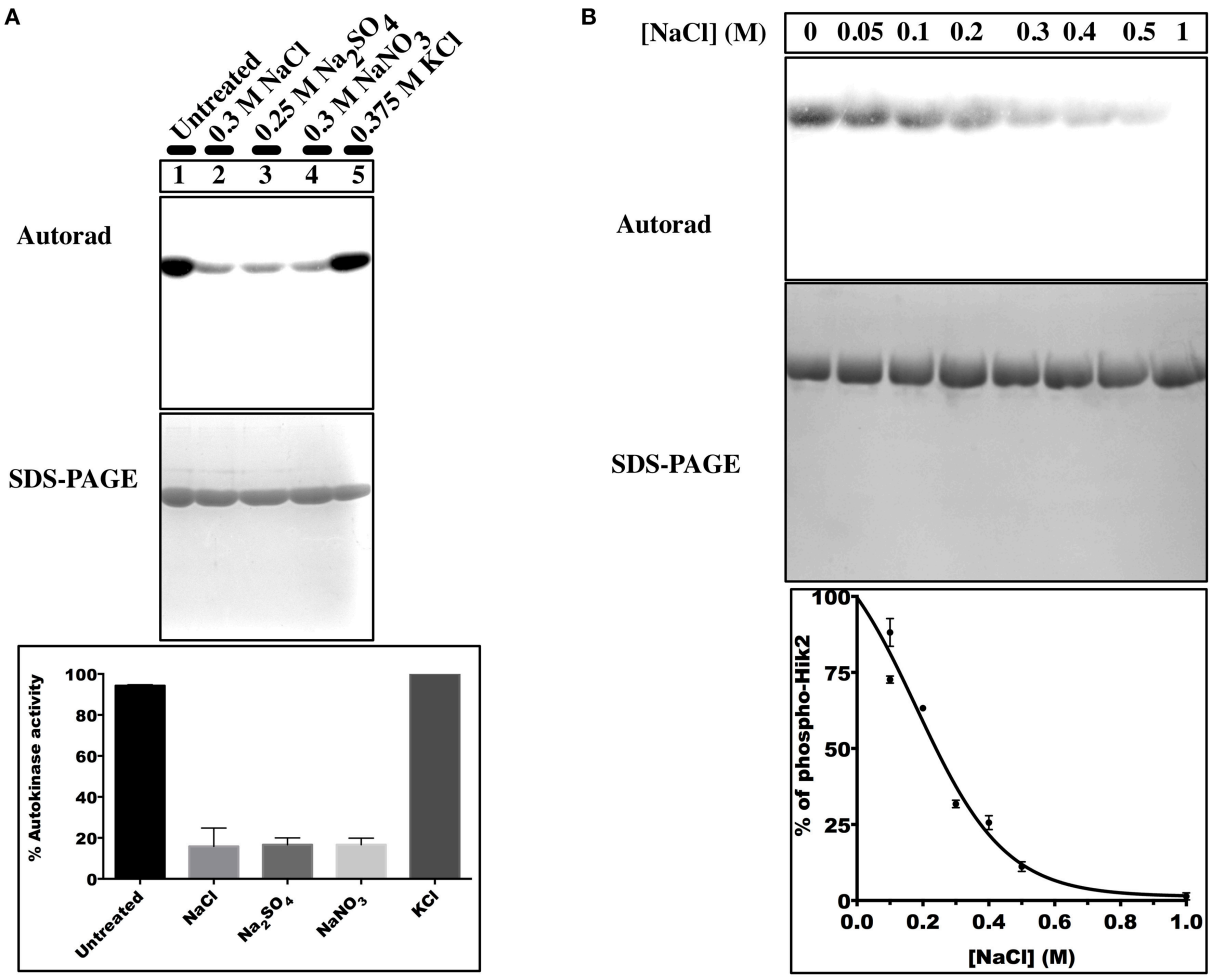
**Effects of different salts on the autophosphorylation activity of Hik2**. **(A)** Lane 1, untreated sample; lane 2, treated with 0.3 M NaCl; lane 3, treated with 0.25 M Na_2_SO_4_; lane 4, treated with 0.3 M NaNO_3_; lane 5, treated with 0.375 M KCl. **(B)** Concentration-dependent inhibition of Hik2. Data points represent intensity of ^32^P labeling quantified by ImageJ. Each data point is the mean of three measurements ± S.E.

Histidine kinases catalyze transfer of only the γ-phosphate from an ATP molecule to their conserved histidine residue. To test whether Hik2 autophosphorylates as a typical histidine kinase, the recombinant and purified Hik2 was assayed for autokinase activity in the presence of [γ-^32^P]ATP and [α-^32^P]ATP. Figure 2, lane 2 shows that Hik2 remained unlabeled when incubated with [α-^32^P], however, it was heavily labeled with ^32^P upon incubation with [γ-^32^P]ATP (Figure 2, lane 3), suggesting robust autophosphorylation activity in Hik2. Hik2 has been suggested to act as an osmosensor (Paithoonrangsarid et al., 2004). We therefore tested whether Hik2 could directly sense salts. Hik2 was incubated with water (control), NaCl, Na_2_SO_4_, NaNO_3_, or KCl (at final concentrations of 0.3, 0.25, 0.3, and 0.375 M, respectively) in the presence of 2.5μCi of [γ-^32^P]ATP. Figure 3A, lane 1 shows that Hik2, in the absence of salt, is autokinase active. However, when it was treated with NaCl (lane 2), Na_2_SO_4_ (lane 3) or with NaNO_3_ (lane 4), the autophosphorylation activity of Hik2 was decreased by up to 75% compared to the untreated protein. Interestingly, KCl did not inhibit the autophosphorylation activity of Hik2 (lane 5). This suggests that the Na^+^ ion, but not the Cl^−^ ion, is responsible for suppressing the autokinase activity of Hik2. In a dose-response curve, inhibition of 50% autophosphorylation activity of Hik2 was seen at 0.25 M of NaCl (Figure 3B). It has been found that treatment of *Synechocystis* sp. PCC 6803 in 0.5M NaCl reduced its growth rate by 50%, and 0.3M NaCl was sufficient to elicit induction of salt tolerance genes that are under the control of Rre1 (Marin et al., 2003). Therefore, our result of 0.3M NaCl inhibiting the activity of Hik2 (Figures 3A,B) is likely to be physiologically relevant.

### Characterization of the nature of phosphoamino group of Hik2

**Figure 4:**
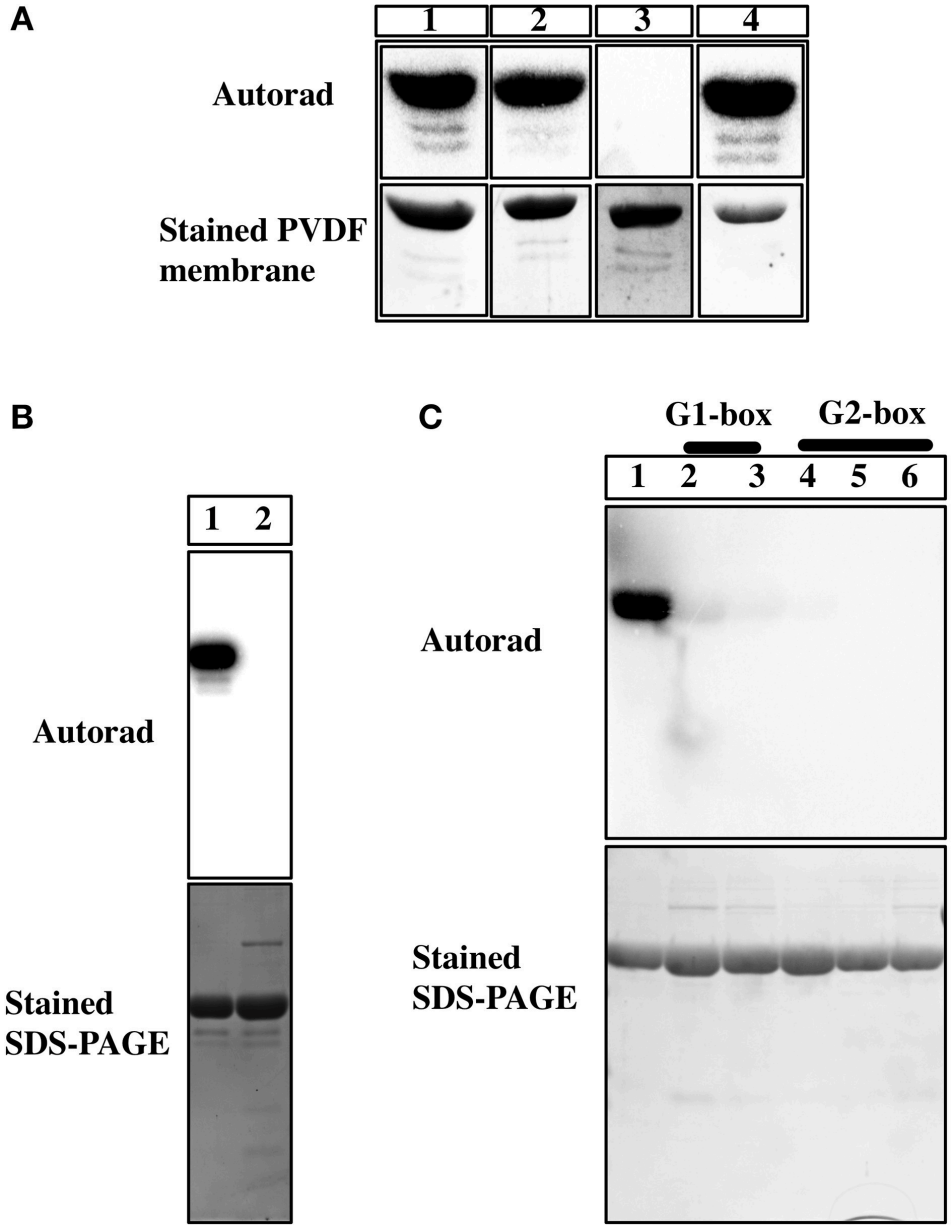
**Characterization of phosphoamino group of Hik2**. **(A)** Autoradiograph of the Acid-base stability assay. Lane 1, untreated Hik2; lane 2, treated with 50mM Tris-HCl (pH 7.4); lane 3, with 1 M HCl; lane 4, with 3 M NaOH. **(B)** Effects of His^185^Q mutagenesis on the autokinase activity of Hik2. Autoradiograph of the autokinase assay is presented. Lane 1 shows the wild-type Hik2 protein. Lane 2, His^185^Q mutant protein. **(C)** Effects of G1 or G2 box mutations on the autokinase activity of Hik2. Autoradiograph of the protein gel from the phosphorylation assay. Lane 1, wild type Hik2 protein; lane 2, G1-box mutant Gly^359^A; lane 3, G1-box mutant Gly^361^A; lane 4, G2-box mutant Gly^386^A; lane 5, G2-box mutant Gly^388^A; lane 6, G2-box mutant Gly^390^A.

In order to understand the nature of phosphoamino acid in Hik2, we used an acid-base stability assay and mutagenesis studies. Phosphorylations on serine and threonine residues are stable in acidic condition, but are labile under alkaline condition. Conversely, phosphorylation on basic residues (histidine, arginine, or lysine), as in histidine kinases, are acid labile but stable under basic condition (Attwood et al., 2007). Phosphorylations on acidic residues, such as aspartate or glutamate, are susceptible to both acid and base hydrolysis (Attwood et al., 2011). We therefore employed an acid-base stability assay to confirm the nature of phosphoamino acid in Hik2. Figure 4A, lane 1 shows that the ^32^P on untreated Hik2 (control) was relatively stable. Figure 4A, lane 2 shows that the ^32^P on Hik2 was relatively stable at pH 7.4 (neutral), at 55°C for 2.5 h. Figure 4A, lane 3 shows that the ^32^P on Hik2 was completely hydrolysed upon incubation in 1M HCl, (acidic condition) at 55°C for 2.5 h. Figure 4A, lane 4 shows that the ^32^P on Hik2 was relatively stable when incubated in 3M NaOH (basic condition), at 55°C for 2.5 h. This behavior of the Hik2 phosphoamino acid is consistent with phosphorylation on a histidine residue, as in sensor histidine kinases. We next mutated the conserved histidine (His^185^) to glutamine. Figure 4B, lane 2 shows that the His^185^Q mutation completely abolished ^32^P labeling of Hik2. Our results are consistent with the single autophosphorylation site of Hik2 being His^185^.

The ATP-binding domain of histidine kinases contains conserved motifs essential for autokinase activity. These include G1 and G2 boxes, which have the characteristic glycine signatures “DxGxG” and “GxGxG,” respectively. The conserved glycine residues in G1 and G2 boxes of Hik2 were identified by sequence alignment; they were then individually substituted to alanine residues in order to establish their role in the autokinase activity of Hik2. Figure 4C shows that the wild-type protein becomes autophosphorylated; however, substitution of the first or second conserved glycine residues in the G1-box abolished the autophosphorylation activity of Hik2 (Figure 4C, lanes 2 and 3). Similarly, substitution of any of the conserved glycine residues within the G2-box completely abolished the autophosphorylation activity of Hik2 (Figure 4C, lanes 4, 5, and 6).

### Pull-down assay shows that Hik2 interacts with Rre1 and RppA

**Figure 5:**
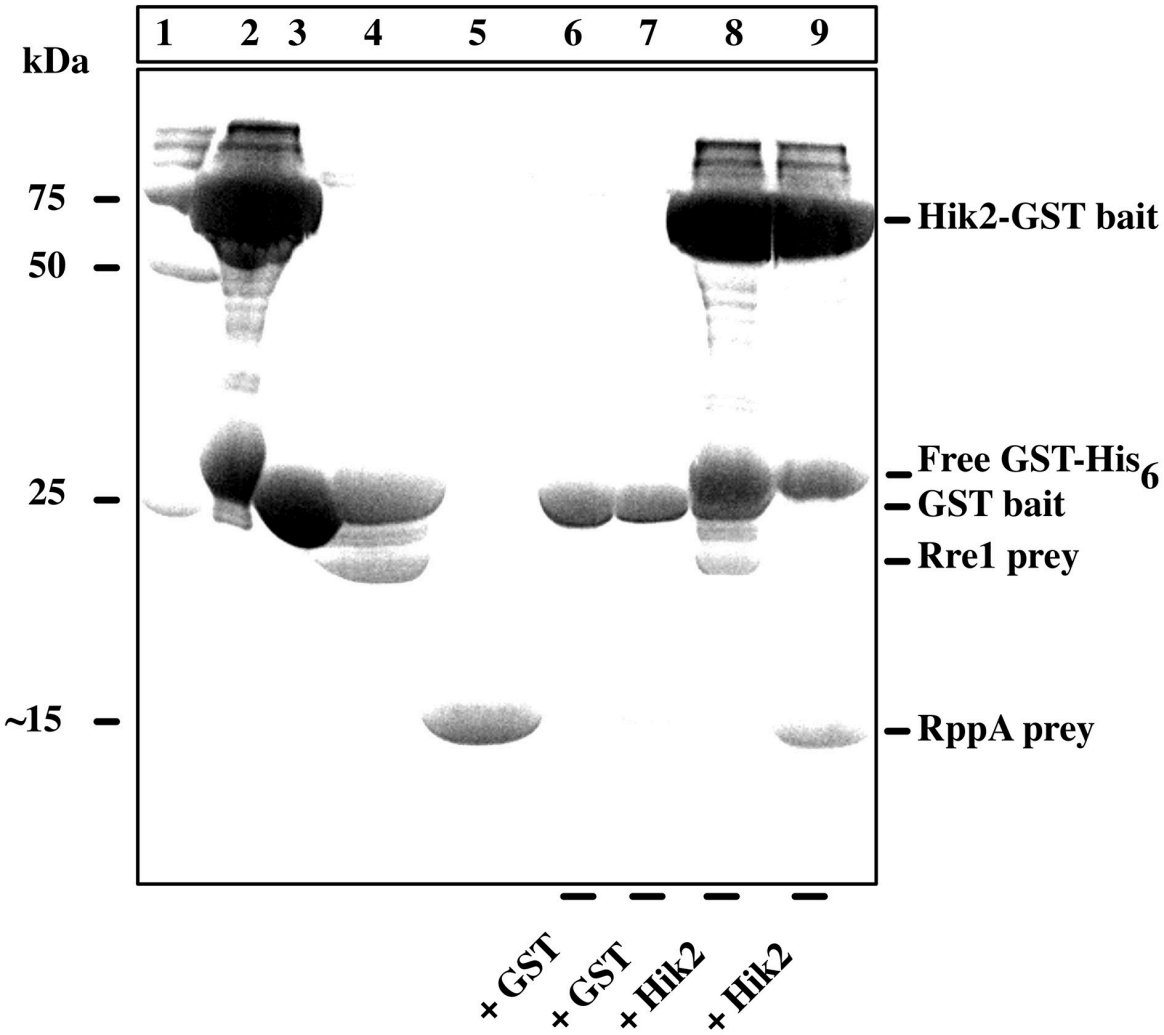
**Interaction of Hik2 with Rre1 and RppA in a GST-based pull-down assay**. Coomassie-stained SDS-PAGE. Lane 1, protein molecular weight markers in kDa; lane 2, over-expressed and purified Hik2-GST bait; lane 3, over-expressed and purified control GST bait; lane 4, over-expressed and purified Rre1 prey; lane 5, over-expressed and purified RppA prey; lanes 6–9 show the products of the pull-down assay. The bait used in each assay is indicated at bottom of the lane. Hik2-GST bait pulls down Rre1 (lane 8) and RppA (lane 9), while the control bait GST does not pull-down either response regulator (lanes 6 and 7, respectively). The positions of the molecular weight markers are indicated on the left. The positions of bait and prey proteins are indicated on the right.

A GST-based pull-down assay was performed to validate the earlier report (Sato et al., 2007) of Hik2 interactions with Rre1 and RppA in a yeast two-hybrid assay. The result in Figure 5 shows that the bait Hik2 protein pulls down prey proteins Rre1 and RppA (Figure 5, lanes 8 and 9, respectively). However, in the control pull-down assay, where GST was used as bait, prey proteins Rre1 and RppA were not co-purified with Hik2 (Figure 5, lanes 6 and 7, respectively), suggesting specific Hik2-Rre1 and Hik2-RppA interactions.

### Phosphotransfer kinetics of Hik2 reveals preferential phosphotransfer to Rre1 and RppA response regulators

**Figure 6:**
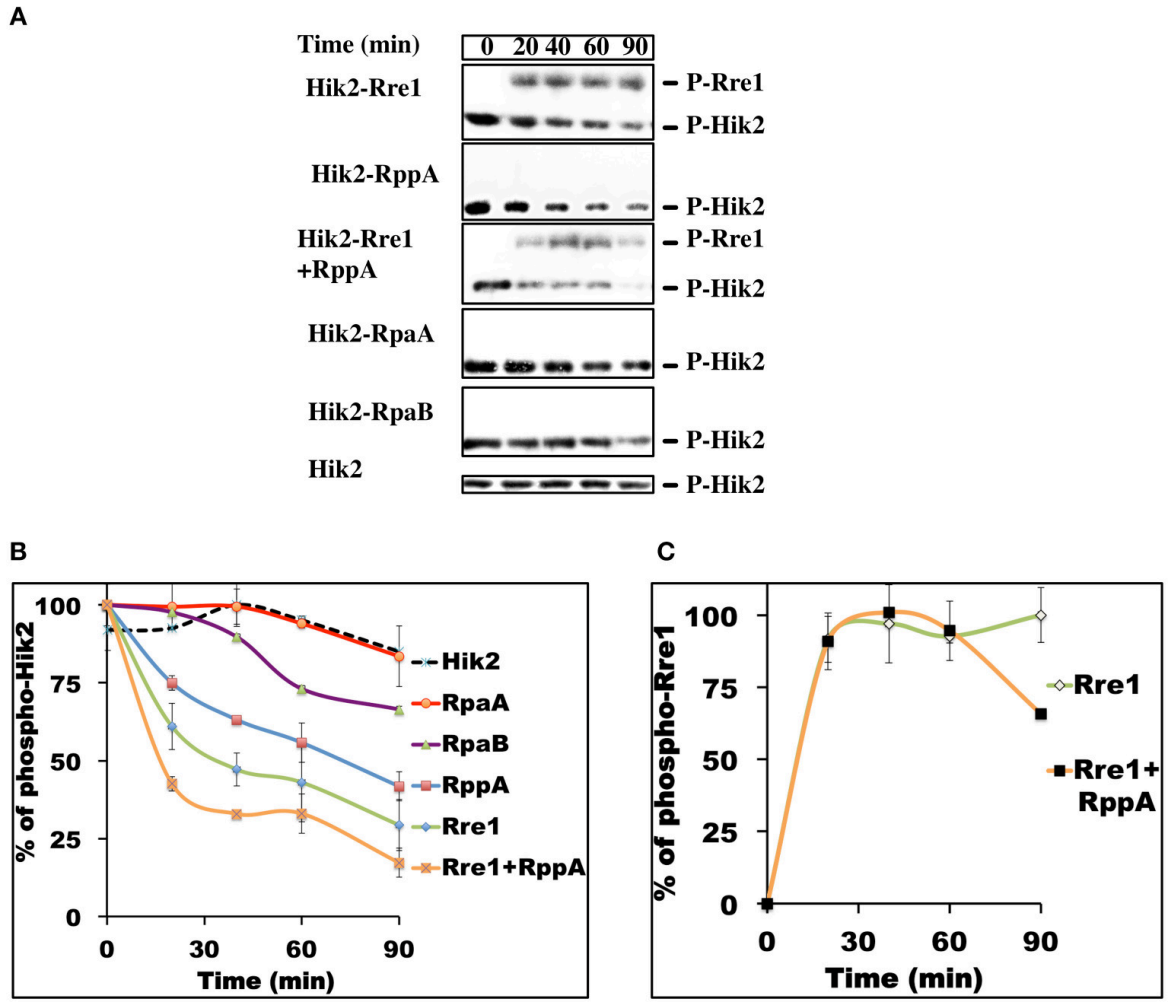
**Time-course of phosphotransfer from Phospho-Hik2 to full-length Rre1, RppA, and RpaA response regulators**. **(A)** Autoradiograph of ^32^P labeling. **(B)** Kinetics of phosphotransfer from P-Hik2 to response regulators. **(C)** Phosphorylation of Rre1F. The assay was repeated at least three times, with similar results obtained in each case. Each data point is calculated from band intensity from the autoradiograph, using ImageJ, and the percentage of activity was plotted as a function of time, in minutes. Error bars indicate standard error of the mean value from three experiments.

In bacteria, individual two-component systems are insulated from each other for minimal cross-talk and faithful signal transmission (Skerker et al., 2008). Co-evolving amino acid residues in sensor kinases and response regulators establish this separation, which is manifested as preferential phosphotransfer kinetics from the sensor kinase to its cognate response regulator. Cognate kinase-response regulator pairs therefore exhibit faster phosphotransfer kinetics than non-cognate pairs (Skerker et al., 2008). The phosphotransfer analysis of Hik2 was performed with the full-length response regulators (Figures 6A,B) as in (Laub et al., 2007; experimental section). Results in Figures 6A,B showing kinetics of dephosphorylation of phospho-Hik2 indicate that Rre1 dephosphorylates phospho-Hik2 the fastest when compared to RppA, RpaA, and RpaB. Hik2-RppA exhibited the second fastest phosphotransfer kinetics. Furthermore, when Rre1 and RppA were mixed together, they dephosphorylated phospho-Hik2 at a much higher rate than each on its own. Moreover, the result presented in Figures 6A,B shows differences in the stabilities of the phosphoryl groups on Rre1 and RppA. The phosphate group on Rre1 is relatively stable, while RppA loses its phosphate group rapidly. We therefore could not detect phosphate groups on RppA in our experimental condition. Our inability to detect a phosphoryl group on RppA is likely to be the result of a rapid autodephosphorylation reaction in RppA, such as in some response regulators (Laub et al., 2007). The phosphotransfer kinetics toward RppA (Figures 6A,B) were therefore inferred from the loss of phosphates from the sensor kinase, Hik2. RpaA and RpaB exhibited slower kinetics comparing to Rre1 and RppA, suggesting that they are less likely to be response regulators of Hik2 under this experimental condition. Phosphoryl group on Rre1 is less stable in the presence of RppA (Figure 6C).

## Discussion

The work presented in this study shows that the full-length recombinant Hik2 protein of *Synechocystis* sp. PCC 6803 purified from *E. coli* becomes autophosphorylated *in vitro*, as predicted on the basis of sequence information and comparison with other histidine sensor kinases. Interestingly, when Hik2 was treated with NaCl (Figure 3A, lane 2), its autophosphorylation activity was inhibited. Further examination of salt sensing activity of Hik2 led us to determine that it responds specifically to Na^+^ ions (Figure 3A). We found that Cl^−^ ions do not affect the autokinase activity of Hik2 (Figure 3A, lane 5). Interestingly, a chimeric sensor kinase, made up of the sensor domain of Hik2 and the kinase domain of Hik7, has been reported to respond to Cl^−^ ions *in vivo* (Kotajima et al., 2013). Our finding, in contrast, shows that the kinase activity of full-length Hik2 protein is modulated by Na^+^ ions (Figure 3), and not by Cl^−^ ions. The reason for an apparent inconsistency between the results presented here with purified proteins (Figure 3) and those reported for whole cells (Kotajima et al., 2013) may be that additional, unspecified interactions occur *in vivo*.

Bacterial cells exposed to high salt concentrations have to cope with lower water potential and higher ionic potential, which can otherwise be toxic to cellular metabolism (Los et al., 2010). Sodium ions, when present in excess in the cytoplasm, compete for potassium-binding sites in proteins and lead to the malfunction of proteins by destabilizing their tertiary structure. In cyanobacteria, an increase of sodium ion concentration in the cell is linked to an efflux of potassium ions. Furthermore, salt stress has a marked inhibitory effect on photosynthesis. Treatment of cyanobacteria with high concentrations of NaCl results a 40% decrease in the amount of the D1 protein of the photosystem II reaction center complex, thus leading to a decrease in the rate of photosystem II mediated oxygen evolution (Sudhir et al., 2005). It is therefore vital that cyanobacteria contain robust regulatory system(s) to achieve salt and osmotic homeostasis. Indeed, to date, four multi-functional sensor histidine kinases—Hik10, Hik16, Hik33, Hik34—have been proposed to sense salt, while Hik2 has been suggested to function as an osmosensor (Paithoonrangsarid et al., 2004). The inhibitory effect of salt on Hik2 autophosphorylation (Figure 3) supports the possibility that Hik2's osmosensing properties are a direct result of its sensitivity to salt. It is therefore likely that Hik2 is a genuine salt sensor, and that this has been overlooked in the earlier study on Hik2 salt sensing (Paithoonrangsarid et al., 2004). We do not yet know how Hik2 senses salt. The GAF sensor domain in Hik2 may bind Na^+^, as in the case of some Na^+^ sensors (Cann, 2007; Biswas and Visweswariah, 2011). Na^+^ ions directly modulating the kinase domain, as in the case of the bacterial osmosensor EnvZ (Wang et al., 2012), is another possibility.

It remains to be determined whether Hik2 senses and responds to other regulatory signals. The Hik2 homolog in chloroplasts, CSK, binds quinone (Puthiyaveetil et al., 2013). This raises the prospect of Hik2 also sensing plastoquinone (PQ) redox state, and thereby regulating photosynthesis genes. If this is indeed the case, Hik2 would qualify as a multi-sensor kinase.

Histidine kinases contain a conserved kinase domain, consisting of DHp and CA subdomains. The phosphorylation site of a histidine kinase is located within the first helix of the DHp domain. We confirmed the phosphorylation site of Hik2 to be His^185^ by using an acid-base stability assay and site-directed mutagenesis (Figures 4A,B). The CA domain contains conserved motifs, G1 and G2 boxes, which are characterized by “DxGxG” and “GxGxG” respectively. The CA domain is essential for binding the ATP molecule and for priming the γ-phosphate of ATP for a nucleophilic attack by the conserved histidine residue that is located within the H-box (Conley et al., 1994). In particular, conserved glycine residues in the G2-box facilitate the flexibility of the ATP-lid, which controls the entry and the release of the ATP-Mg^2+^ complex and the ADP-Mg^2+^ complex, respectively (Marina et al., 2001). Consequently, mutations within the G1 or G2 boxes disrupt the structure of the nucleotide-binding pocket, and thereby impair the autophosphorylation of histidine kinases. Indeed, mutation within the G1 or G2 box for several histidine kinases abolishes their autokinase activity (Gamble et al., 1998; Chen et al., 2009). Along these lines, substitution of any of the conserved glycine residues with alanine in the G1 or G2 box of Hik2 abolishes its autophosphorylation activity (Figure 4C).

The second step in a TCS is the phosphotransfer reaction from the conserved histidine residue of the histidine kinase to an aspartic acid residue in the response regulator. The phosphotransfer reaction between cognate sensor-response regulator pairs has favored kinetics, and these can be used to identify functional partners (Laub et al., 2007; Skerker et al., 2008). The phosphotransfer kinetics shown in Figure 6 illustrate that Hik2 has the highest phosphotransfer activity toward Rre1, followed by RppA (Figures 6A,B). These findings are consistent with the earlier yeast-two hybrid study (Sato et al., 2007) and with the pull-down assay results presented in Figure 5, and further confirm that Rre1 and RppA are cognate response regulators of Hik2. Our results (Figure 6) also rule out RpaA and RpaB as functional partners of Hik2 in cyanobacteria.

The precise functional role and target genes of Rre1 in cyanobacteria are not yet clear, though Rre1 has been suggested to regulate osmotic responsive genes (Paithoonrangsarid et al., 2004). The homolog of Rre1 in red algal plastids, Ycf29, binds to phycobiliprotein genes in low light, where it is then likely to activate their expression (Minoda and Tanaka, 2005). The notion that Rre1 can have different effects at salt/osmotic/light-stress-responsive target genes is consistent with the fact that it is a NarL-type response regulator. NarL-type response regulators can act as activators as well as repressors of transcription depending on the nature and location of their binding sites in their target genes (Maris et al., 2002).

In cyanobacteria and chloroplasts, the redox state of the PQ pool controls transcription of chloroplast genes that encode reaction-center proteins of photosystem II and I, initiating a long-term acclimatory process known as photosystem stoichiometry adjustment (Pfannschmidt et al., 1999). In cyanobacteria, this process has been suggested to involve the RppA response regulator (Li and Sherman, 2000). Our phosphotransfer analysis (Figure 6) further supports this possibility. Interestingly the sensor kinase RppB, found in the same operon as RppA, turned out to have no role in the regulation of photosynthesis genes (Li and Sherman, 2000). Sensor kinases other than RppB have therefore been proposed to work with RppA in photosystem stoichiometry adjustment in cyanobacteria (Li and Sherman, 2000). We suggest Hik2 is the cognate sensor of RppA in this regulatory pathway. Since the number of sensor kinases tends to be lower than that of response regulators in bacteria, more than one response regulator is likely to partner with a given kinase (Laub and Goulian, 2007). The functional role of CSK, the chloroplast homolog of Hik2 (Puthiyaveetil et al., 2008), together with the results presented here and elsewhere (Sato et al., 2007), supports Hik2 being the sensor that acts on Rre1 and RppA transcription factors to regulate photosynthesis genes as part of the mechanism of photosystem stoichiometry adjustment in cyanobacteria. The same Hik2-Rre1 system acting on a different set of target genes may also underlie the salt/osmotic tolerance in cyanobacteria. Salt stress, like light quality changes, induces photosystem stoichiometry adjustment in cyanobacteria, and a common sensory system has been suggested to govern these two responses (Murakami et al., 1997). Our work identifies this shared signaling system, with the Hik2 sensor kinase as the hub integrating both salt and redox signals and the Rre1 and RppA response regulators forming its bifurcated arms. A similar, bifurcated quinone redox signaling pathway has been proposed to connect regulation of photosynthetic reaction center stoichiometry with regulation of the relative light-harvesting antenna size of photosystem I (PS I) and photosystem II (PS II) during light state 1-state 2 transitions(Allen, 1995; Allen and Nilsson, 1997; Li and Sherman, 2000).

**Figure 7:**
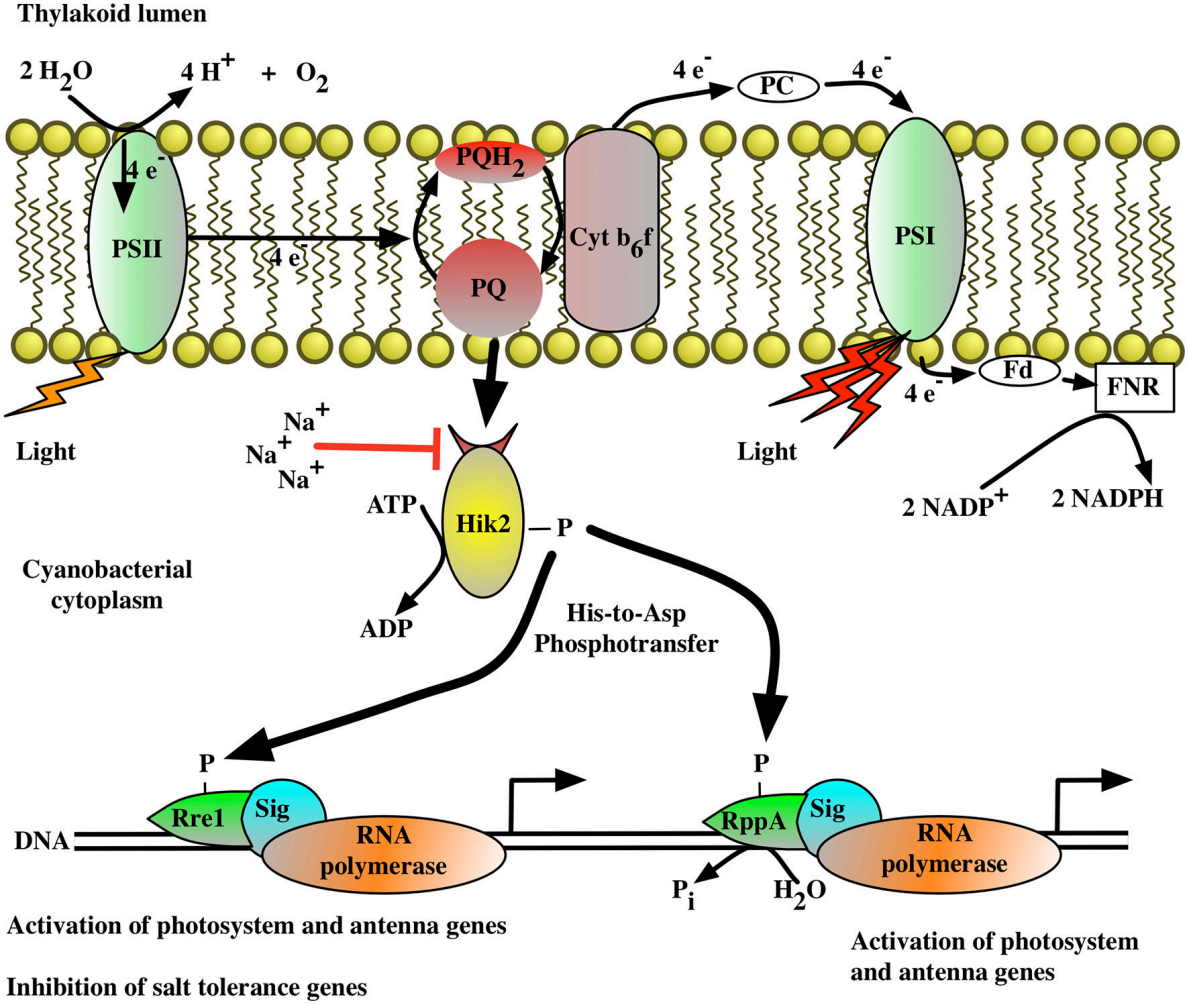
**The proposed signal transduction pathway for the Hik2-based two-component system in cyanobacteria**. The autophosphorylation activity of Hik2 is regulated by signals from the photosynthetic electron transport chain and by sodium ions (Na^2+^). The red arrow indicates an inhibitory effect. A black arrow indicates activation. Autophosphorylated (active) Hik2 transfers phosphoryl groups to Rre1 and RppA. Phosphorylated Rre1 and RppA activate genes encoding the photosynthetic machinery. In addition, the phosphorylated form of Rre1 acts as a negative regulator for salt/osmotic tolerance genes. Under high salt/osmotic condition, Na^2+^ inhibits the autophosphorylation of Hik2. Phospho-Rre1 becomes dephosphorylated, which in turn removes the repression of salt/osmotic tolerance genes.

Figure 7 presents a working model of transcriptional control by the Hik2-based two-component signal transduction system in cyanobacteria. In this model (Figure 7), the activated Hik2 autophosphorylates and transfers phosphoryl groups to Rre1 and RppA. Phospho-Rre1 activates genes coding for phycobilisomes. Phospho-RppA regulates genes for photosystems, thereby balancing the distribution of excitation energy driving electron transfer between PS II and PS I by means of photosystem stoichiometry adjustment. Our scheme also posits that phospho-Rre1 represses salt/osmotic tolerance genes, a suggestion consistent with the fact that Rre1 belongs to the NarL-type family of response regulators, which act as both activators and repressors of transcription at different target genes (Maris et al., 2002). Upon salt and/or hyperosmotic signal, the activity of Hik2 is inhibited (Figure 3, lanes 2–4); Rre1 and RppA therefore remain in their unphosphorylated states. As a result, Rre1 can no longer act as a repressor of salt/osmotic tolerance genes, in turn releasing the repression on their transcription.

While our results demonstrate that Hik2 is a multifunctional sensor histidine kinase, they do not rule out the possibility of Hik2 having additional inputs and outputs, as yet uncharacterized. A central and co-ordinating position of Hik2 in diverse cellular control circuits would be consistent with the indispensability of the *Hik2* gene for cyanobacterial growth and viability (Li and Sherman, 2000; Paithoonrangsarid et al., 2004; Ashby and Houmard, 2006).

## Author contributions

IMI performed the experimental work and devised experimental strategies; SP advised and supervised; JFA posed questions and outlined the investigation; all three authors contributed to interpretation of the data, discussion, conclusions, and to writing the manuscript.

### Conflict of interest statement

The authors declare that the research was conducted in the absence of any commercial or financial relationships that could be construed as a potential conflict of interest.
